# Supplementary figures and images for: Identification of a genome-specific repetitive element in the Gossypium D genome
Source: PeerJ. 2020 Jan 3;8:e8344. doi: 10.7717/peerj.8344 (PMC6944119; doi:10.7717/peerj.8344)

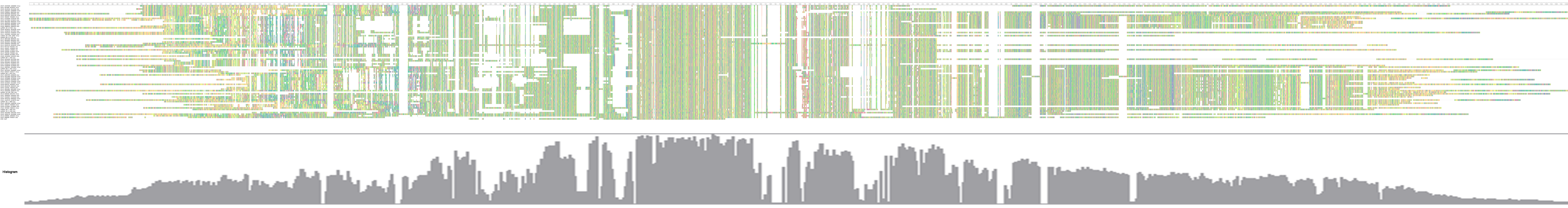

Supplement: Figure S1 [file peerj-08-8344-s006.png]
