# Supplementary material for: Identification of a genome-specific repetitive element in the Gossypium D genome
Source: PeerJ. 2020 Jan 3;8:e8344. doi: 10.7717/peerj.8344 (PMC6944119; doi:10.7717/peerj.8344)
Supplement: Doc S1 [file peerj-08-8344-s007.docx]

1. The script for batching sequence extracting form genome or database:

#!/usr/bin/perl

use strict;

use warnings;

##############################

# Name: batch.intercept.pl

# Description: Batch get and intercept sequence from database than output to certain file

# Usage: perl $0 <database> <info> <out>

# Version: 1.0

# Author: Lu Hejun luhejun@foxmail.com

# Date: 2015-02-07 10:41:41

##############################

if(@ARGV!=3)

{

print "\n\#Please Input As Follow\#\:\n";

print "perl $0 <database> <info> <out>\n";

print "\#The Format of everyline in <info> file must be\:\nseqname\tstar\tend\tdef\nOr\:\nseqname";

exit;

}

my ($seq,$seq_title)=();

($seq,$seq_title)=each_seq($ARGV[0]);

my $select_seq="";

open INF,"$ARGV[1]";

open OUT,">$ARGV[2]";

foreach (<INF>) {

if ($_=~/(\w+)\t(\w+)\t(\w+)\t(\w*)/) {

my $seqname=$1;

my $star=$2;

my $end=$3;

my $def=$4;

print OUT "$seq_title->{$seqname} $star-$end $def\n";

my $select_seq=substr($seq->{$seqname},$star-1,$end-$star+1);

my @splitline=split(//,$select_seq);

$select_seq="";

for(my $i=0;$i<@splitline;$i++)

{

$select_seq.=$splitline[$i];

$select_seq.="\n" if(($i+1)%50==0);

}

print OUT $select_seq."\n";

}

elsif ($_=~/(\w+)$/) {

my $seqname=$1;

my $select_seq=$seq->{$seqname};

print OUT "$seq_title->{$seqname}\n$select_seq.\n";

}

else {

print "\n\#Please Input As Follow\#\:\n";

print "perl $0 <database> <info> <out>\n";

print "\#The Format of everyline in <info> file must be\:\nseqname\tstar\tend\tdef\nOr\:\nseqname";

exit;

}

}

close INF;

close OUT;

sub each_seq

{

my $file=shift;

open FILE,"$file" || die "Cannot open the file '$file'.\n";

my $title=();

my ($seq,$seq_title)=();

while(<FILE>)

{

chomp;

if($_ ne "" && $_=~/^\>/)

{

$title=(split(/\s+/,$_))[0];

$title=~s/^\>//;

$seq_title->{$title}=$_;

}

elsif($_ ne "")

{

$seq->{$title}.=$_;

}

}

close FILE;

return ($seq,$seq_title);

}

1. This script for illustrate the synteny block between two or more segments:

#!/usr/bin/perl

use strict;

##########################################

# Name: SynBlockPlot.pl

# Description: Illustrate syntenic relation between chromosomes

# Usage: perl

# Version:1.0

# Author:Hejun Lu luhejun@foxmail.com

# Date: April 7th, 2017

##########################################

use Getopt::Long;

use File::Basename qw(basename dirname fileparse);

use IO::File;

use Data::Dumper;

use Pod::Text;

use SVG;

use Cwd qw(abs_path getcwd);

my $bin = dirname(abs_path($0));

my (@len, @syn, $out, @gap);

GetOptions(

'l=s'=>\@len,

's=s'=>\@syn,

'g=s'=>\@gap,

'o=s'=>\$out,

)|| &help;

if(!defined($out))

{

print <<" Usage End.";

############################

Description:

Illustrate Syntenic Relation Between Chromosomes

Usage:

perl $0 -l Chr1.len -l Chr2.len -l Chr3.len -s Chr1.Chr2.syn -s Chr2.Chr3.syn -o new.svg

-l infile must be given

-s infile must be given

-o out.svg must be given

h Help document

The Format of everyline in <len> file must be\:

Chr length

The Format of everyline in <syn> file must be\:

Chr1 start end Chr2 start end

############################

Usage End.

exit;

}

my $svg = SVG->new(width => 1200, height =>1200);

my (%style, %rect, );

&iniSvg();

my $unit = 500000;

my @chr_spc_x = (12, 4, 6, 12, 4, 6) ;

my $chr_spc_y = 65;

my $chr_height = 8;

my $gap_ratio = 0.5;

my $curve = 30;

my %chr_style = %style;

my $green="#00BB00";

my $yellow="#EEEE00";

my $gray="#BBBBBB";

my $pink="#FF6EB4";

my $red="#EE0000";

my $blue="#0000BB";

my $purple="#8E236B"; #"#B23AEE";

my $light_blue="#97FFFF";

my $orange="#CD950C";

my $light_green="#7FFF00";

my $green2="#76EEC6";

my $purple2="#4B0082";

my $purple4="#FF00FF";

my $blue2="#1E90FF";

my $blue3="#8DB6CD";

my $purple3="#7A67EE";

my $color1="#8D00CD";

my $color2="#0067EE";

my @blackground = ("#FAF9DE", "#CCFFCC", "#E3EDCD", "#DCE2F1", "#C7EDCC", "#EBEBE4", "#108AC6", "");

my @colors= map {'#E8E8E9'} (0..500000);

my @genome_colors = ('#BF368E', '#00803E', '#F4A6C8', '#BF368E', '#00803E', '#F4A6C8');

my @star_x;

my @star_y;

my @all_genome;

for my $idx (0..$#len){

$star_x[$idx] = 40;

$star_y[$idx] = 40+$chr_spc_y*$idx;

push @all_genome, &getGenomeLen($len[$idx]);

}

my %genome_art = (genome => $all_genome[0],

star_x => $star_x[0],

star_y => $star_y[0],

chr_spc_x => $chr_spc_x[0],

chr_spc_y => $chr_spc_y,

height => $chr_height,

style => \%style,

txt_pos => 'up');

$genome_art{style}{fill} = $genome_colors[0];

&drawGenome(%genome_art);

for my $idx (0..$#syn){

$genome_art{genome} = $all_genome[$idx+1];

$genome_art{star_x} = $star_x[$idx+1];

$genome_art{star_y} = $star_y[$idx+1];

$genome_art{chr_spc_x} = $chr_spc_x[$idx+1];

$genome_art{txt_pos} = $idx == $#syn ? 'down' : 'other';

$genome_art{style}{fill} = $genome_colors[$idx+1];

&drawGenome(%genome_art);

my @syn_region = &readSyn($syn[$idx]);

my @pos = &caluSynPos(\@syn_region, @all_genome[$idx, $idx+1],

$idx==0 ? 's' : $idx==$#syn ? 'e' : '1');

&drawSyn(\@pos);

}

open my $OUT, '>', $out;

print $OUT $svg->xmlify();

sub drawSyn

{

my ($pos) = @_;

for my $ref(@{$pos}){

my %syn_style = %style;

$syn_style{"fill-opacity"} = 1;

$syn_style{"stroke-opacity"} = 0;

#$syn_style{fill} = $colors[&fetchNum($$ref[1])-1];

$syn_style{fill} = '#E8E8E9';

$svg->path(%{$$ref[0]}, style => \%syn_style);

}

}

sub caluSynPos

{

my ($syn, $up, $down, $staus) = @_;

my @res;

for my $ref (@$syn){

my @up_x = map { $_/$unit + $up->{$$ref[0]}{x}} @{$ref}[1,2];

my @down_x = map { $_/$unit + $down->{$$ref[3]}{x}} @{$ref}[4,5];

my $up_y = $up->{$$ref[0]}{y} + $up->{$$ref[0]}{height}*0.5 ;

my $up_cont_y = $up_y + $curve;

my $down_y = $down->{$$ref[3]}{y} + $down->{$$ref[3]}{height}*0.5 ;

my $down_cont_y = $down_y - $curve;

my $point = { 'd' => "M $up_x[0] $up_y ".

"C $up_x[0] $up_cont_y $down_x[0] $down_cont_y $down_x[0] $down_y ".

"H $down_x[1] ".

"C $down_x[1] $down_cont_y $up_x[1] $up_cont_y $up_x[1] $up_y ".

"Z"

};

push @res, [$point, $$ref[0], $$ref[-1]] ;

}

return (@res);

}

sub drawGenome

{

my %p = (

genome => '',

star_x => 0,

star_y => 0,

height => 0,

style => '',

txt_pos => '', # med up down

@_,

);

&caluChrPos($p{genome}, $p{star_x}, $p{chr_spc_x});

&drawSeriesRect(%p);

&drawSeriesTxt(%p);

}

sub caluChrPos

{

my ($genome, $star_x, $chr_spc_x) = @_;

my @pos = ([$star_x], );

for my $chr (sort {&fetchNum($a)<=>&fetchNum($b)} keys %{$genome}){

$genome->{$chr}{x} = $pos[-1][0];

push @{$pos[-1]}, $genome->{$chr}{len}/$unit;

$genome->{$chr}{width} = $pos[-1][1];

push @pos, [$pos[-1][0]+$pos[-1][1]+$chr_spc_x];

}

pop @pos;

}

sub drawSeriesRect

{

my %p = (@_);

my %chr = %rect;

$chr{height} = $p{height};

$chr{y} = $p{star_y};

$chr{rx} = '4';

$chr{ry} = '4';

for my $chr (keys %{$p{genome}}){

my $ref = [$p{genome}->{$chr}{x}, $p{genome}->{$chr}{width}];

$p{genome}->{$chr}{y} = $p{star_y};

$p{genome}->{$chr}{height} = $p{height};

$chr{x} = $$ref[0];

$chr{width} = $$ref[1];

my %style = %{$p{style}};

my %n_sty = %style;

$style{'fill'} = 'white';

if ($chr{width}<13){

$chr{rx} = '0';

$chr{ry} = '0';

}

$svg->rectangle(%chr, style=>\%style);

my %n_chr = %chr;

$n_chr{y} = $p{star_y} + $p{height}*$gap_ratio*0.5;

$n_chr{rx} = '1';

$n_chr{ry} = '1';

$n_chr{height} = $p{height}*$gap_ratio;

$n_sty{"fill-opacity"} = '1';

$n_sty{"stroke-width"} = '0';

$svg->rectangle(%n_chr, style=>\%n_sty);

}

}

sub drawSeriesTxt

{

my %p = ( txt_pos => '', @_);

for my $chr (keys %{$p{genome}}){

my $ref = [$p{genome}->{$chr}{x}, $p{genome}->{$chr}{width}];

my $chr_num = &fetchNum($chr);

my %txt;

if ($p{txt_pos}=~/(up)|(down)/){

$txt{x} = $$ref[0]+$$ref[1]/2;

$txt{y} = $p{txt_pos} eq 'up' ?

$p{star_y} - $p{height}/2 :

$p{star_y} + $p{height}*5/2 + 4;

$txt{'text-anchor'} = 'middle' ;

}elsif($chr=~/old/){

next if $chr_num%10;

$txt{x} = $$ref[0]+$$ref[1]/2;

$txt{y} = $p{star_y} + $p{height}*5/2 + 4;

}else{

$txt{x} = $$ref[0]+$$ref[1]+5;

$txt{y} = $p{star_y} + $p{height}*3/2;

$txt{'text-anchor'} = 'start';

}

###ori $txt{-cdata} = $chr;

###add

$txt{-cdata}=$chr_num;

###add

$svg->text(%txt);

}

}

sub readSyn

{

my $file = shift;

open my $IN,'<',$file;

my @res;

while(<$IN>){

chomp;

my ($a_chr, $as, $ae, $b_chr, $bs, $be, $lend) = split;

push @res, [$a_chr, $as, $ae, $b_chr, $bs, $be, $lend];

}

return (@res);

}

sub getGenomeLen

{

my $IN = IO::File->new(shift);

my %res;

while(<$IN>){

chomp;

my ($chr, $len) = (split);

$res{$chr}{len} = $len ;

}

return \%res;

}

sub iniSvg

{

%style = (

'stroke' => 'black',

'fill' => 'white',

'stroke-width' => '1',

'stroke-opacity' => '1',

'fill-opacity' => '0',

);

%rect = (

x => 0,

y => 0,

width => 0,

height => 0,

);

}

sub fetchNum

{

my $name = shift;

my $num = $name=~/(\d+)/ ? $1 : die"cannot find a num";

$num=~s/^0+//;

return $num;

}

sub getGapPos

{

my $file = shift;

my $cutoff = shift;

$cutoff||=5000;

my %seq = &getSeq($file);

my %pos;

foreach my $chr (keys %seq)

{

my $chrseq = $seq{$chr};

while( $chrseq=~/([nN]{1000,})/g)

{

my $len = length($1);

my $end = pos($chrseq);

my $start = $end-$len+1;

push @{$pos{$chr}},[$start,$end];

}

}

return (\%pos);

}

sub getSeq

{

my $file=shift;

my %chr;

$/=">";

open FA,$file;

<FA>;

while(<FA>)

{

chomp;

my($head,$seq)=split/\n+/,$_,2;

my $id =(split/\s+/,$head)[0];

next if $id=~/dom/ || $id=~/old/ || $id=~/onti/ || $id=~/chrC/;

$seq=~s/\n+//g;

$seq=~s/>$//;

$chr{$id}=$seq;

}

close FA;

$/="\n";

return (%chr);

}
