# Supplementary material for: Identification of a genome-specific repetitive element in the Gossypium D genome
Source: PeerJ. 2020 Jan 3;8:e8344. doi: 10.7717/peerj.8344 (PMC6944119; doi:10.7717/peerj.8344)
Supplement: Doc S2 [file peerj-08-8344-s008.docx]

1. The sequence of the representative *ICRd* motif

>Chr05_50639971_50641791

CTTCAACGTACTCCACTGTAACCTTAGGGAGGTAAAATCCGCCATCTTCGATCTACTCCACTACTGCTTAGGGAGACAAGATCTGAAATCTTCAATCTATTCGCTGCTGCCCAGGGAAGTAGAATTACCGGCTTCAATGTGCTCCACTGTAACCACAGGGAGGTAAAATTCATCATCTTTGATCTGCTCCACTACTGCTTGGGGAGACAAGATCTAAAATCTTTTAATCTATTCCACTGCTGACCAGGGAAGTAGAATTTCTGGCTTCAATGTACTCCACTGCAACCTCAAGGAGGTAAAATTCACCATCTTTGATCTGCTCCACTGTCTATGCAGGAAGGCAAGATCTGAAATCTTTAATCTATTTCACTGCTGACCAGGGAAGTAGAATTTCTGGCTTCAATGTACTCCACTGCAACCTCAGGGAGGTAAAATTCACCATCTTTGATCTGCTCCGCTGTTTATGCAGGAAGGCAAGATCTGAATCTTTATCTATTCCACTGCTGCCCAGGGAAGTAGAATTTCTGGCTTTAATGTACTCCACTGCAACTTCAGGGAGGTAAAATCCACCATCATTGATCTGCTCCGCTGTTTATGCAGGAAGGCAAGATCTGAAATCTTTAATCTATTCCACTGCTGCCCAGGGACGTAGAATTTCTGGCTTCAATGTACTCCACTGCAACTTCAGGGAGGTAAAATCCACCATCTTTGATCTGCTCCGCTGTCTATGCAGGAAGGCAAGATCTGAAATCTTTAATCTATTCCATTGCTGCCCAGGGAAGTAGAATTACTGGCTTCAATGTACTCCACTGCAACTTCATGGAGGTAAAATCCGCCATCTTCGATCTGCTCCACTACTGCTTAGGGAGACAAGACCTGAAATCTTCAACCTGCTCCACTGTCTCGAGGGAGGCGAGGTTGGTGTCTTTGATCTGCTTCACTTTCGATGCAGGAAGGCAAGATCTGTTATCATCACTGGTCTGTTCTCTGGGGAACATGACCTGTATAATGAACTTTATGAACCTAATTATGCCTAGTGATTAGGATGCCATGATCAGAATGAATCAAATACTCCTAACTAGACATGTATGAATGATATTTGAATGAATGCAGAATGTCATGAAAATGATATTTTAACGCTTGGGTTATTATTACTCCAAGTTTATTAAGGGTTCATCACTAATGTGTTATAACACCTTCTTGCTCAGCTGGCGTCTCCAAAGAAACACTTAGTCAGATTGCCCCACACTGTAAACCTCTAAGTTTGATCCACTGGGACGCAAAATTTGAACCATCTTTTTCCCGTTGTAACTCAAGGGTAGAAAGATTTGACCTTTTTTCAATCCTCTGCTATCACAATTCGAGGATAGAGGATGTGAAACTTTTTGGTCCCTTACACCATTCCCAGGGTGTTGTACCAAATGCTCATGTACAAATGAAGAACTTTTCTCCAAGAACAACTTCTTCTTGTTATTCGGTGATCATTGCTTGCTTGTTCATTGAAGCTTTGTCACCAACACGACATCTTGCCATTTTGATCAATCGATGTTTTAGACAGTAAAAACTCAAAGGGATAGTCTTTAATTTAGACTCTTCTTTATCAGATTTCCAACTTGGTGCGTTCTGAACAATAGTCCTGTTTAAGGTTCCTATATTATTCAGAGACTTCTAGAGTAATATGCAAAACTTCCCTTGTGAAAATTTTATTAGTCCATTAATCATTGTTCTAATGCAACATGCTTGCAAAAAGGTCATAACAATGGGTAGGAATAAAATTGATTTAGAGCATGGCTCTGAAATGAATAGATTCTCGAAGATAAT

Tandem repeat region (TR): 1-911

Basic unit: 1-118, 118-251, 252-384, 358-515, 516-648, 649-781, 782-911

Conservative sequence (CS): 912-1821

2. Query in GIRI Repbase

We queried the 1.8 kb repetitive sequence in GIRI Repbase with the selection of all the organisms as database, the query result showed this repeat sequence do not found any matching in other organisms except two partial matches with two LTR-Gypsy annotated from *G. raimondii* with the identity lower than 80% respectively.

Table S1. Query result of the repetitive sequence in GIRI Repbase

| Name | length | From | To | Name | length | From | To | Identity | Class |
| --- | --- | --- | --- | --- | --- | --- | --- | --- | --- |
| Chr05_50639971_50641791 | 1821 | 9 | 313 | Gypsy-51_GR-I | 12206 | 11447 | 11794 | 0.75 | LTR/Gypsy |
| Chr05_50639971_50641791 | 1821 | 314 | 1821 | Gypsy-9_GR-I | 12270 | 10499 | 12004 | 0.74 | LTR/Gypsy |

This sequence highly repeats in *G. raimondii* genome with more than 80% identity and more than 80% matching ratio (Table S1), while the query result in GIRI showed partial matching and low identity and revealed this repetitive sequence have not yet been annotated in GIRI.
